# Genome-wide association and biological pathway analysis for milk-fat composition in Danish Holstein and Danish Jersey cattle

**DOI:** 10.1186/1471-2164-15-1112

**Published:** 2014-12-15

**Authors:** Bart Buitenhuis, Luc LG Janss, Nina A Poulsen, Lotte B Larsen, Mette K Larsen, Peter Sørensen

**Affiliations:** Department of Molecular Biology and Genetics, Center for Quantitative Genetics and Genomics, Aarhus University, Blichers Allé 20, P.O. Box 50, DK-8830 Tjele, Denmark; Department of Food Science, Aarhus University, Blichers Allé 20, P.O. Box 50, DK-8830 Tjele, Denmark

## Abstract

**Background:**

The milk fat profile of the Danish Holstein (DH) and Danish Jersey (DJ) show clear differences. Identification of the genomic regions, genes and biological pathways underlying the milk fat biosynthesis will improve the understanding of the biology underlying bovine milk fat production and may provide new possibilities to change the milk fat composition by selective breeding. In this study a genome wide association scan (GWAS) in the DH and DJ was performed for a detailed milk fatty acid (FA) profile using the HD bovine SNP array and subsequently a biological pathway analysis based on the SNP data was performed.

**Results:**

The GWAS identified in total 1,233 SNPs (FDR < 0.10) spread over 18 chromosomes for nine different FA traits for the DH breed and 1,122 SNPs (FDR < 0.10) spread over 26 chromosomes for 13 different FA traits were detected for the DJ breed. Of these significant SNPs, 108 SNP markers were significant in both DH and DJ (C14-index, BTA26; C16, BTA14; fat percentage (FP), BTA14). This was supported by an enrichment test. The QTL on BTA14 and BTA26 represented the known candidate genes *DGAT* and *SCD*. In addition we suggest ACSS3 to be a good candidate gene for the QTL on BTA5 for C10:0 and C15:0. In addition, genetic correlations between the FA traits within breed showed large similarity across breeds. Furthermore, the biological pathway analysis revealed that fat digestion and absorption (KEGG04975) plays a role for the traits FP, C14:1, C16 index and C16:1.

**Conclusion:**

There was a clear similarity between the underlying genetics of FA in the milk between DH and DJ. This was supported by the fact that there was substantial overlap between SNPs for FP, C14 index, C14:1, C16 index and C16:1. In addition genetic correlations between FA showed a similar pattern across DH and DJ. Furthermore the biological pathway analysis suggested that fat digestion and absorption KEGG04975 is important for the traits FP, C14:1, C16 index and C16:1.

**Electronic supplementary material:**

The online version of this article (doi:10.1186/1471-2164-15-1112) contains supplementary material, which is available to authorized users.

## Background

Genetic analysis of bovine milk fat has shown that there is heritable variation underlying the biosynthesis of bovine milk-fat (e.g. [1]). Some fatty acids (FA) are synthesized *de novo* in the mammary gland and are showing a moderate to high heritability, like e.g. short and medium chain saturated FA. In contrast, long chain FA are derived from blood lipids, which are originating from the diet and endogenously produced lipids. These long chain FA have, however, low to moderate heritabilities [1–3]. Identification of the genes underlying the genetic variation of the biosynthesis of milk fat would enhance the understanding of the biology of the fatty acid biosynthesis. Polymorphisms in major genes like diacylglycerol O-acyltransferase 1 (*DGAT1*) and stearoyl-CoA desaturase (*SCD*) have a large influence on the milk fat composition [4–7], however it has been shown that the biosynthesis of milk fat is a complicated process regulated by many genes [8]. This is supported by a genome wide association study (GWAS) that has been published on milk FAs in Dutch dairy cattle showing that medium chain and unsaturated FA are strongly influenced by *DGAT* and *SCD* respectively, but other regions also showed significant association [9].

Poulsen et al. [10] showed that there was a considerable difference between breeds in the milk fatty acid composition. The Danish Jersey (DJ) cows were characterized by higher levels of saturated short chain FA compared to the Danish Holstein (DH), whereas the DH had higher content of unsaturated C18 FA [10]. Furthermore, it was suggested that the content of C14:1 and C16:1 in the milk was mainly genetically regulated [10]. The differences between breeds could be due to the difference in their genetic background. It is likely that the same genes are involved in the biosynthesis of milk FA, but some processes may be pronounced due to the genetic background resulting in a different milk fat profile. Screening the populations using a GWAS approach, results in the underlying genomic regions and genes influencing the trait of interest [9]. Furthermore, the results of the GWAS can be used to search for genetic differences and similarities between different breeds as well as searching for the biological pathways involved in the trait of interest [11].

The aim of this study was two-fold 1) perform a GWAS using the bovine HD SNP array in both the DH and DJ breed in order to identify genomic regions in both breeds and identify similarities and differences between the breeds in the genetic regulation of milk FA and 2) identify the biological pathways underlying the FA biosynthesis based on the SNP markers.

## Results

### Correlation between traits based on SNP markers

**Figure 1:**
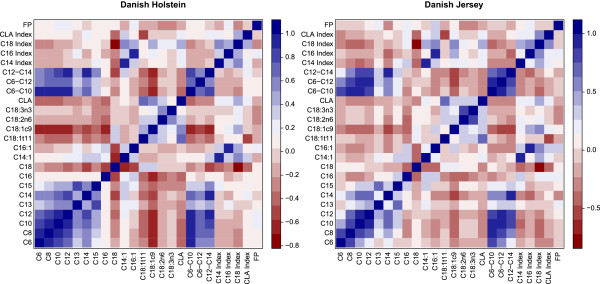
**Genetic correlation estimated based on the t-values from the association study between the different individual fatty acids and groups of fatty acids in the Danish Holstein breed and Danish Jersey breed.** The red colour represent a negative correlation, the blue colour represents a positive correlation.

**Figure 2:**
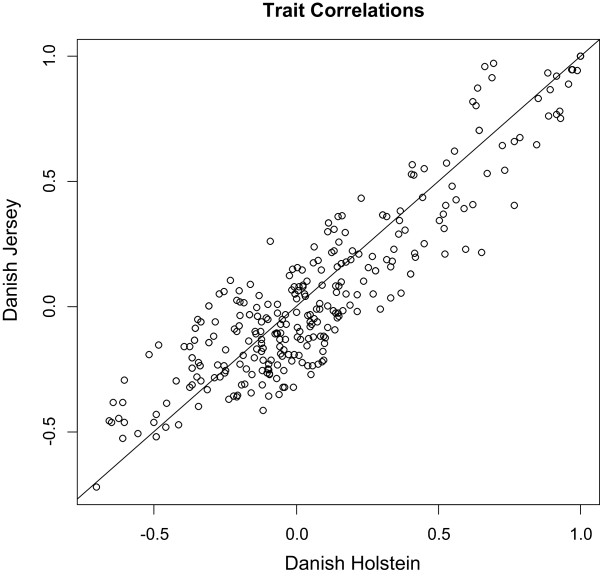
**Correlation between the correlations for individual fatty acids and groups of fatty acids within Danish Holstein (x-axis) and Danish Jersey (y-axis).**

The t-values of the association analysis were the basis to estimate the genetic correlation between the FA traits within breeds (Figure 1). There was a high correlation between the saturated short to medium chain FA (C6:0, C8:0, C10:0, C12:0, C13:0 and C14:0) for the Holstein breed. This high correlation was also observed between the groups of saturated short to medium chain FA (C6-C10; C6-C12, C12-C14) and the individual short to medium chain saturated FA. The long chain saturated FA C16:0 and C18:0 had a low or negative correlation with the long chain saturated FA. Furthermore, there was a negative correlation between the unsaturated FA and the saturated FA. Comparing the DH to the DJ one can see that the correlation patterns within breed were similar (Figure 1) and that there was a high correlation between the trait correlations observed in the DH compared to the trait correlations observed in the DJ (Figure 2).

### QTL detected for the Danish Holstein

The results for the significant SNP markers for the DH are presented in Additional file 1: Table S1. In total 1,233 significant SNP markers have been identified, spread over 18 different chromosomes for nine FA traits (C6:0, C8:0, C14:1, C16:1, CLA, C6-C10, C14 index, CLA index, fat percentage (FP)). For C6:0, 32 significant SNP markers were spread over BTA9 (26 SNPs: 22,833,168 bp to 26,284,743 bp), BTA12 (2 SNPs), and BTA25 (4 SNPs: 16,440,279 bp to 16447599). For C14:1, 83 significant SNPs were detected distributed over BTA5 (2 SNPs), BTA7 (1 SNP assigned to *GRIA1*), BTA8 (10 SNPs of which six SNPs were assigned to *SYK*), BTA12 (4 SNPs) and BTA26 (21 SNPs of which two SNPs could be assigned to *ZFYVE27*, three SNPs to *CRTAC1*, three SNPs to *DNMBP*, and seven SNPs could be assigned to *SCD*). *SCD* explained 12.7% of the total variance. For C16:1, 29 significant SNPs were distributed over two chromosomes: BTA8 (2 SNPs), BTA14 (27 significant SNPs in the range from 1,588,879 bp to 2,764,862 bp). Among the significant SNPs on BTA14 some could be assigned to known genes; *ARHGAP39*, *CYHR1*, *CPSF1*, *DGAT1*, *SMPD5*, *GRINA*, *LOC786966*, and *FAM83H*). *DGAT1* explained 7.8% of the total variance. For CLA, two SNPs were significant on BTA17. These SNPs were assigned to *FAT4*. For C6-C10, 11 significant SNPs were detected on BTA9 (22,839,756 bp to 26,284,743 bp). Two SNPs could be assigned to *SOGA3*. For C14 index, 398 significant SNPs were detected of which 368 on BTA26 (24,022,413 bp to 41,656,807 bp), of these 368 SNPs, 167 SNPs could be assigned to 41 genes (Additional file 1: Table S1). The SNP with the highest –log_10_(P-value) was assigned to *SCD* explaining 20.9% of the total variance. For CLA index, seven significant SNPs were detected on BTA16 of which five could be assigned to *PTPRC*. For FP, in total 628 significant SNPs were detected. On BTA2, 11 SNPs were detected and assigned to PARD3B. On BTA11, nine SNPs were detected. However, the majority of the significant SNPs were detected on BTA14 (573 SNPs in a range from 1,427,669 bp to 8,682,547 bp). The significant SNP markers could be assigned to 30 different genes. *DGAT1* was among these 30 genes. The SNP assigned to DGAT1 explained 22.8% of the total variance (Additional file 1: Table S1).

### QTL detected for the Danish Jersey

The results for the significant SNP markers for the DJ are presented in Additional file 2: Table S2. In total 1,122 significant SNP markers have been identified, spread over 26 different chromosomes for 13 FA traits (C10:0, C12-C14, C13:0, C14:0, C14 index, C15:0, C16:0, C18:0, C18:1n9, C18:2n6, C18:3n3, CLA and FP). For C10:0, five significant SNPs were identified of which three could be assigned to genes (BTA13: *GSS*, *UQCC*, and BTA26: *SEC31B*). On BTA5, five significant SNPs were identified for C13:0 and on BTA17, two SNPs were significant. All five SNPs on BTA5 were assigned to *ACSS3* explaining 11.8% of the total variance*.* For C12-C14, three SNPs on BTA15 were significant of which two SNPs were assigned to *CADM1* (BOVINEHD1500025093, BOVINEHD1500025092). For C14 index, 308 significant SNPs were spread over five chromosomes. Most SNPs were detected on BTA26 (189) in the range of 4.5 Mb to 23.7 Mb. SNPs in this range could be assigned to 32 genes of which *SCD* is one (Additional file 2: Table S2). *SCD* explained 66.5% of the total variance. Furthermore three SNPs were detected on BTA14, 10 SNPs on BTA15, two SNPs on BTA18, and one SNP on BTA23. For C15:0, 78 SNPs were significant, spread over nine chromosomes. The majority of these SNPs (47) were located on BTA5. The top SNPs were assigned to *ACSS3*, explaining 17.7% of the total variance. On BTA20, six significant SNPs were significant and on BTA28, 13 significant SNPs were detected. For C16:0, 33 significant SNPs were significant of which 25 SNPs were located on BTA14, and eight SNPs were located on BTA27. The SNP assigned to *DGAT1* on BTA14 explained 12.5% of the total genetic variance. For C18:0, 80 SNPs were significant of which 51 SNPs were located on BTA10, and 29 SNPs were located on BTA27. Among the significant SNPs on BTA10 some SNPs could be assigned to *TDP*, *KCNK13*, *TTC7B*, *CASC4*, and *CTTDSPL2*, while on BTA27 some SNPs could be assigned to *SUPT3H*, and *RUNX2*. For C18:1n9, three significant SNPs were located on BTA15, of which two could be assigned to *CADM1*. For C18:2n6, 191 significant SNPs were spread over 17 different chromosomes. The majority of the SNPs were located on BTA9 (43), BTA13 (40), BTA24 (19) and BTA4 (16), but only a few SNPs could assigned to a gene. For C18:3n3, 12 SNPs were significant of which 11 were located on BTA8. No SNPs were assigned to genes. For CLA three significant SNPs were located on BTA10, which were all assigned to *KCNH5*. For FP, 320 SNPs were found to be significant. The majority of the SNPs were located on BTA11 (126) and BTA14 (164). Significant SNPs on BTA11 could be assigned to CAPN14, GALNT14, and LCLAT1. On BTA14 *DGAT1* is among the genes assigned to SNPs. The SNP assigned to *DGAT1* on BTA14 explained 12.5% of the total genetic variance.

### Comparison between breeds

**Figure 3:**
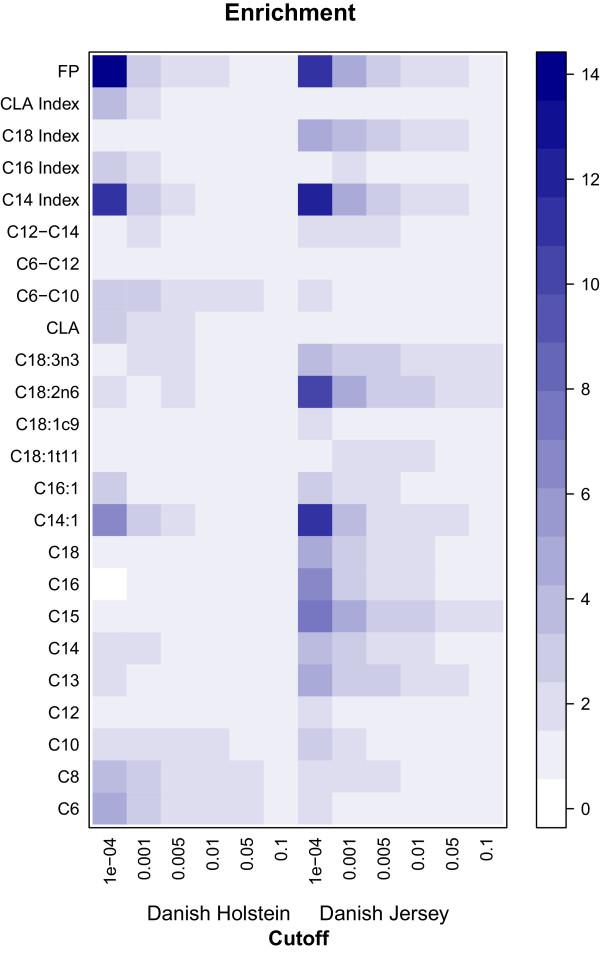
**Enrichment plot for the different fatty acids and groups of fatty acids for both the Danish Holstein and Danish Jersey breeds at different cut-off levels for the P-values.** The colour shade from white to dark blue indicates the fold change in enrichment for each trait.

**Figure 4:**
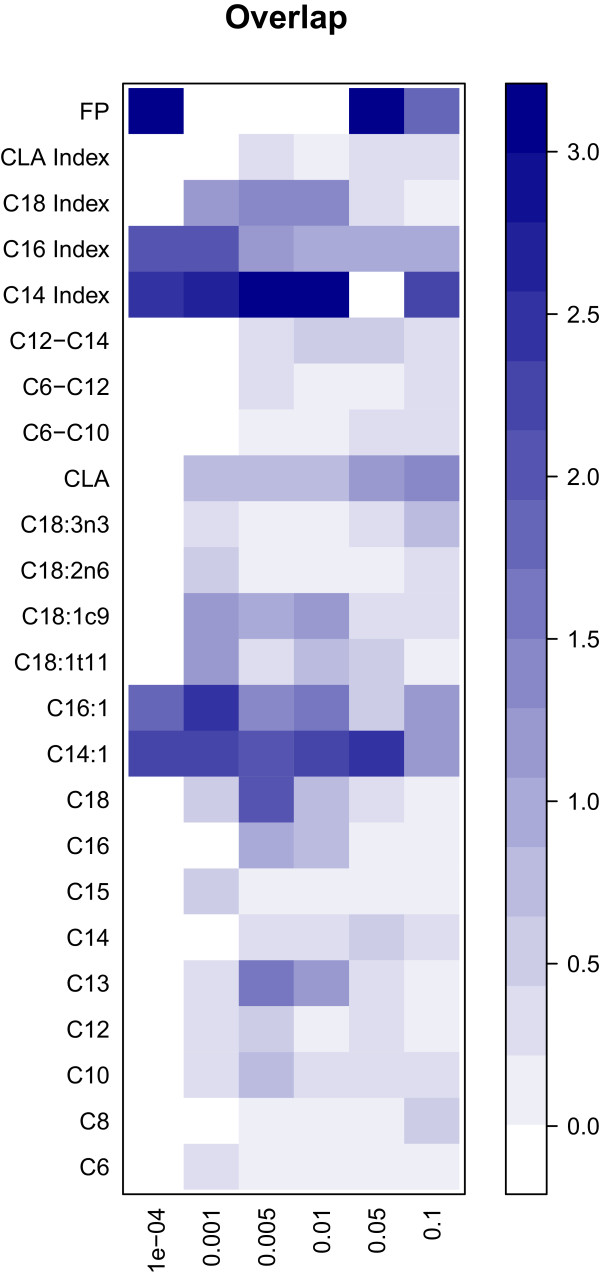
**Overlap of significant SNP markers between Danish Holstein and Danish Jersey breed for the different fatty acids and groups of fatty acids.** The figure represents the P-values for the overlap. Dark blue represents significant overlap (P < 10^-3^) between the Danish Holstein and Danish Jersey breeds.

Figure 3 presents the enrichment of associated SNPs at five different cut-off values for the P-values obtained from the GWAS within each breed. For the traits FP, and C14 index there was a 14 fold higher number of significant SNP markers than expected for both the DH and the DJ at a cut-off value of 10^-4^. C14:1 showed a 5 fold higher number of significant SNP markers than expected for the DH, whereas for the DJ a 14 fold higher number of significant SNP markers than expected was shown. For DJ, C18:2c6 showed a 12 fold higher number of significant SNP markers than expected. At the cut-off value for the P-values of 10^-4^ it was shown that there was significant overlap in SNP markers between the DH and DJ for the traits FP, C14 index, C14:1, C16 index and C16:1 (Figure 4).

### Biological pathways detected for the Danish Holstein and Danish Jersey breeds

In the results for the biological pathways, we will focus on the traits, which showed which showed significant overlap between DH and DJ in associated SNPs (i.e. FP, C14 index, C14:1, C16 index and C16:1). Furthermore, we only mention the biological pathways for each fatty acid with P < 0.05. For the reader who is interested in the results of the other FA or groups of FA, we refer to Additional file 3: Table S3, which presents the P-values for each biological KEGG pathway for each fatty acid and groups of FA for both the DH and DJ breed.

### Fat percentage

For DH, 22 different KEGG pathways were significant at the P < 0.05 level, whereas 10 KEGG pathways were significant for DJ (Additional file 4: Table S4). The two breeds had the two most significant pathways in common: KEGG04975 Fat digestion and absorption (P < 0.0039 for DH and P < 1e^-4^ for DJ) and KEGG00830 Retinol metabolism (P < 0.0045 for DH and P < 6e^-4^ for DJ).

### C14 index

For C14 index, 26 KEGG pathways were significant for the DH, whereas 19 KEGG pathways were significant for DJ at the P < 0.05 level (Additional file 5: Table S5). In total, six KEGG pathways were in common between DH and DJ: KEGG00350 Tyrosine metabolism (P < 0.0131 for DH and P < 0.0218 for DJ), KEGG00360 Phenylalanine metabolism (P < 0.0076 for DH and P < 0.0037 for DJ), KEGG00400 Phenylalanine, tyrosine and tryptophan biosynthesis (P < 0.0375 for DH and P < 0.0057 for DJ), KEGG00790 Folate biosynthesis (P < 0.0372 for DH and P < 0.0022 for DJ), KEGG05332 Graft-versus-host disease (P < 0.0299 for DH and P < 0.0353 for DJ), KEGG05340 Primary immunodeficiency (P < 0.0077 for DH and P < 0.0015 for DJ).

### C14:1

For C14:1, 18 KEGG pathways were significant for DH, whereas 20 KEGG pathways were significant for DJ at the P < 0.05 level (Additional file 6: Table S6). There is however little overlap between breeds. The two breeds had two pathways in common: KEGG00350 Tyrosine metabolism (P < 0.0447 for DH and P < 0.02 for DJ) and KEGG00400 Phenylalanine, tyrosine and tryptophan biosynthesis (P < 0.0375 for DH and P < 0.0057 for DJ).

### C16 index

Out of the 32 significant KEGG pathways for DH and 25 significant KEGG pathways for DJ (Additional file 7: Table S7), only KEGG00760 Nicotinate and nicotinamide metabolism (P < 0.0097 for DH and P < 0.0172 for DJ) and KEGG0465 Natural killer cell mediated cytotoxicity (P < 0.0355 for DH and P < 0.0499 for DJ) were in common between the two breeds.

### C16:1

For C16:1, 29 KEGG pathways were significant for DH and 19 KEGG pathways were significant for DJ at the P < 0.05 level (Additional file 8: Table S8). However, only KEGG05332 Ether lipid metabolism pathway (P < 0.0012 for DH and P < 0.0164 for DJ) was in common between the two breeds.

## Discussion

In this study we have performed a GWAS in two independent breeds (DH and DJ) with the intention to learn more about the underlying genetic architecture of individual FA in bovine milk. A previous study based on the same data showed that the DH and the DJ breeds have a different fatty acid profile of the milk [10]. It has been shown that some of the FA are more regulated by the environment (e.g. long chain FA), while the regulation of other FA (short and medium chain FA) is more influenced by genetics [1]. As the FA differing between the DH and DJ belong to both groups [10], it would be of interest to compare the genetic architecture of the two breeds to identify differences and similarities between the breeds in the genetic regulation of FA in the milk.

### Genome-wide association comparison to other studies

Recently, a GWA study on Dutch Holstein cattle was published using the 50 k bovine SNP array identifying 54 regions on 29 chromosomes [9]. Ten FA (C6:0, C8:0, C10:0, C12:0, C14:0, C16:0, C18:0, C14:1, C16:1, and CLA) were measured in our study as well as in Bouwman et al. [9]. However, there was little overlap between the QTL found in Bouwman et al. [9] and in our study for these FA. For DH, significant SNP markers were detected for C14:1, C16:1 and CLA on the same chromosomes as detected by Bouwman et al. [9]. The most surprising difference with the results of Bouwman et al. [9] was that in our study we did not detect significant association for C16:1 on BTA26 and for C14:0 on BTA14. Looking in more detail only markers for C14:1 on BTA7 (62.4 Mb-64.2 Mb) and BTA26 (2.5 Mb-41.2 Mb), and markers for C16:1 on BTA14 (0 Mb-26.3 Mb) were overlapping with Bouwman et al. [9]. The SNP markers detected for C10:0, C14:0, C16:0 in DJ had overlap with the study of Bouwman et al. [9] on BTA13 (49.5 Mb-71.5 Mb) and BTA26 (2.5 Mb-41.2 Mb) for C10:0, BTA15 (20.5 Mb-27.0 Mb) for C14:0, and on BTA14 (0 Mb-26.3 Mb), and BTA27 (28.7 Mb-48.3 Mb) for C16:0.

In German Holstein cattle, four main QTL have been detected explaining 46.18% of the estimated breeding value for FP. The four QTL (genes) were located on BTA5 (*EPS8*), BTA14 (*DGAT1*), BTA20 (*GHR*) and BTA27 (*GPAT4*), respectively [12]. In our study, we identified for both the DH and DJ significant markers on BTA11 and BTA14 for FP, but not for BTA5, BTA20 and BTA27.

The main difference between the studies of Bouwman et al. [9] and Wang et al. [12] and our study is 1) the study populations used in Bouwman et al. [9] and Wang et al. [12] are more than four times as big as our study populations. This would mean that the power to detect smaller QTL is larger compared to our study, 2) the studies of Bouwman et al. [9] and Wang et al. [12] used the 50 K bovine SNP array, while our study we used the bovine HD SNP array. An increased number of markers would increase the power to detect a QTL, 3) the significance threshold used in the different studies is different. Bouwman et al. [9] used a threshold of –log(P-value) ≥ 3, while Wang et al. [12] used a threshold of –log(P-value) > 5.88 (Bonferroni P-value < 1.3 × 10^-6^ [12]). The FDR correction for multiple testing indicated a SNP marker to be significant when -log(P-value) ≥ 3.9, in our study. This significance level is between the aforementioned studies. Based on the different significance levels it is expected that our study would find a smaller number of significantly associated SNPs compared to Bouwman et al. [9], and a larger number of significantly associated SNPs compared to Wang et al. [12]. These differences between the studies may be the reason why there is relatively little overlap between studies.

In our study we used the GC method to identify the FA profile in the milk on a relatively small number of animals. We used the GC method was because this is an accurate method to determine the fatty acid content in the milk. To increase the number of animals the GC method is suitable but expensive in use and is therefore less suitable to screen large numbers of animals. An alternative method would be mid-infrared (MIR) spectrometry to assess the detailed FA profile in the milk [13]. In some countries this method has been extended to measure detailed FA profiles on a regular basis and genetic parameters for specific FA based on MIR spectrometry has been published [14].

### Candidate genes located in the QTL

#### DGAT1

The *DGAT1* enzyme plays an important role in triacylglycerol synthesis by catalyzing the esterification of a fatty acyl-CoA to the sn-3 position of a diacylglycerol. The *DGAT1* gene is located on BTA14, and it has been shown that a polymorphism in the *DGAT1* gene explains the QTL for milk yield and composition [15]. In our study, *DGAT1* shows association to C16:1 and FP explaining 7.8 and 22.8% of the total variance, respectively for DH and in DJ, *DGAT1* was associated with C16:0 explaining 12.5% of the total variance. This is in line with the literature, where it has been shown that *DGAT1* is underlying large genetic variation in milk fat composition traits, among these traits were milk FP, C14:0, C16:0, and CLA [7]. Furthermore, it was shown that *DGAT1* is associated with desaturation indexes in Dutch Holstein and Italian Brown cattle [4, 6]. In our study however we did not find any association with *DGAT1* and desaturation indexes.

#### SCD

The *SCD1* enzyme catalyzes the conversion of C10:0 to C18:0 saturated fatty acid into their mono-unsaturated counterparts. The *SCD1* gene is located on BTA26 and is associated with milk fatty acid composition [4–6]. In our study, we have detected associations with *SCD* for C14:1 and the C14 index for DH and for the C14 index for DJ. Especially for the C14 index we found strong association with *SCD* in both breeds, explaining a large part of the total variance, 20.9 and 66.5% respectively. However we did not detect any association with *SCD* and the other indices. Mele et al. [5] and Conte et al. [4] also detected association between *SCD* and C14 index and did not find association to other desaturase indexes in Italian Holstein and Italian brown cattle, respectively. On the contrary, Schennink et al. [6] detected association between *SCD* and six different desaturatase indexes of which *SCD* explained the largest part of the genetic variation for the C14 index (52%). The size of the study of Schennink et al. [6] is much larger compared to our study and the studies by Mele et al. [5] and Conte et al. [4] and therefore has a higher power to detect associations. The association of *SCD* with the C14 index has now been confirmed in three different breeds (Holstein, Italian brown and DJ). As C14:0 is almost solely derived from *de novo* synthesis in the mammary gland it is likely that almost all the C14:1 *c*-9 is synthesized by *SCD* [16].

#### ACSS3

Acyl-CoA synthetase short-chain family member isoforms (*ACSS1* and *ACSS2*; both located on BTA13) have been shown to play a role in activation and intracellular channeling of FA. Gene expression studies have shown that *ACSS2* had greater abundance and up-regulation in mRNA expression than *ACSS1* during lactation in the mammary gland [8]. Furthermore it has been shown that *ACSS2* is located in a chromosomal region associated to C6:0, C8:0, C10:0, C14:1, and C16:1 [9]. In our study, however, we did not detect any association with *ACSS2*, but we detected association on BTA5 between *ACSS3* and C10:0 and C15:0 in DJ. *ACSS3* is related to acetate-CoA ligase activity and the propanoate metabolism playing a role together with *ACSS1* and *ACSS2* in the conversion of propanoyl-CoA via proionyladenylate into propanoate (http://www.genome.jp/kegg-bin/show_pathway?hsa00640). Furthermore the most significant SNP located in *ACSS3* explains 11.8 and 17.7% of the total variance for C10:0 and C15:0 respectively. This makes *ACSS3* a good candidate gene for milk-fat composition.

### Comparison Danish Holstein and Danish Jersey

The genetic correlations between the fatty acid and groups of FA showed the same pattern in both DH and DJ. This is a first indication that the underlying genetics of the regulation of FA in the breeds is similar. The overlap of SNPs associated with the individual FA and groups of FA in the two breeds showed that there was a strong indication that the same causal or linked variants were involved in the regulation of FP, C16 index, C16:1, C14 index and C14:1. In addition, there was also an indication that the same SNPs which showed and association in both DH and DJ were involved C18 index, C18:0, CLA and C13:0 (Figure 4). The fact that the same SNP markers are significant for specific FA in the two breeds, strengthen the suggestion that the FA in these breeds are regulated by the same genes. However, due to the fact that the sample size in this study is relatively low, we cannot state that the non-significant P-values for the overlap for marker between DH and DJ for C6:0 to C12 indicates that there are different genetic mechanisms influencing these FA in the two breeds.

### Biological pathways

So far biological pathways in relation to milk fat production in cattle have been studied by looking at the gene expression of genes known to play a role in the fat metabolism by qPCR [8]. Based on the mRNA expression of 45 genes a network among genes involved in fat synthesis has been developed [8]. By using RNA-seq, it was shown that genes involved in the fat metabolism pathway had high expression in milk somatic cells during transition and peak period of the lactation [17]. These studies show that the production of milk fat in the mammary gland can be divided into five distinct processes: fatty acid uptake, de novo fatty acid synthesis, fatty acid desaturation, fatty acid esterification and milk fat secretion [17].

Previously, it has been shown that gene-set enrichment approaches based on SNP associations is a valuable tool to get additional biological information from a GWA study [18]. In this paper, we have used a statistical modeling approach that evaluates the collective action of sets of SNPs on the trait phenotypes. The approach was used to identify genes and biological pathways to the milk traits.

Based on the results for the biological pathway analysis for FP, C14 index, C16 index, C14:1 and C16:1, it is clear that there is overlap of biological pathways between the DH and DJ. Furthermore, some biological pathways are showing up for multiple traits. However, this is expected as the desaturase indices (C14 index and C16 index) are a function of C14:1 and C16:1, respectively.

The biological pathway ‘Fat digestion and absorption’ KEGG04975 is significant for FP, C14:1, C16 index and C16:1. The pathway describes the fat uptake from blood and the digestion process in the intestine in human (http://www.genome.jp/dbget-bin/www_bget?hsa04975). Genes involved in this pathway (e.g. *CD36*, *AGPAT*, *DGAT*, *FAPB*) are however, also expressed in the mammary gland and play a role in e.g. fatty acid uptake from blood (*CD36*), synthesis of TAG (*AGPAT* and *DGAT*), and intra cellular channeling (*FABP*) [19]. Or in more detail: *CD36* and *FABP* have been categorized as being involved in the fatty acid transport [19], *AGPAT* and *DGAT* have been categorized as being involved in the esterification [19]. These processes are described by KEGG04975 in relation to the fat digestion and absorption in the human intestines. Short- and medium-chain saturated FA and about half the amount of C16:0 are produced *de novo*. A small part of C14:0 and C16:0 in the milk is desaturated to C14:1 and C16:1 by *SCD1* [20]. The long-chain FA and about half of C16:0 are taken up through the diet or can be released from adipose tissues from the cow. The long chain FAs are biohydrogenated into different intermediate products before these are absorbed in the mammary gland in two steps: 1) from the rumen into the blood stream, 2) from the blood stream into the mammary gland. These steps fit well with the biological process described in KEGG04975.

Interestingly, the pathway called ‘Phenylalanine, tyrosine and tryptophan biosynthesis’ (KEGG00400) was among the significant for C14 index and C14:1 in both DH and DJ. Studying the biological process behind this pathway showed that tyrosine can be degraded via a number of steps into fumarate and acetoacetate. Acetoacetate is a ketone body, which can be converted into acetyl-CoA, a precursor for the fatty acid synthesis.

It is clear that several cancer and immune response related pathways were significant for DJ compared to DH. How these biological pathways relate directly to the fatty acid production in the milk is unclear at this stage. Furthermore a few biological pathways like linoleic acid metabolism (KEGG00591), α-linoleic acid metabolism (KEGG00592) are related to the formation of fat, but it is not directly clear how these relate to the C16:0 and C14:0 fatty acid formation. However, the pathways described in human regarding fat are based on knowledge about body fat instead of milk-fat. In general the results of the method showed that even though the method reveals the most likely biological pathway (‘fat digestion and absorption’ KEGG04975), it shows that there are also some considerations to be taken into account: 1) in general cancer and immune related pathways are better annotated compared to other type of pathways as these have a larger focus in research. 2) It is clear that assigning 700 k SNPs to genes on the cattle genome would mean that we will lose some genes to be assigned to the different biological pathway groups. This could be improved by using full sequence information. 3) The significance of the biological pathway groups are based on the t-values corresponding to the SNPs assigned to the genes. Due to the different LD structure between DH and DJ, the t-values can differ for the SNPs between DH and DJ and thereby different biological pathways can be significant for the same trait in the two breeds.

## Conclusion

There was a clear similarity between the genetics of the formation of FA in the milk between DH and DJ. This was supported by the fact that there was substantial overlap between SNPs for FP, C14 index, C14:1, C16 index and C16:1. In addition, genetic correlations between FA showed a similar pattern across DH and DJ. Furthermore the biological pathway analysis suggested that fat digestion and absorption KEGG04975 is important for the traits FP, C14:1, C16 index and C16:1.

## Methods

### Animals

All procedures were performed in accordance with the National Guidelines for Animal Experimentation and the guidelines of the Danish Animal Experimental Ethics Committee. Within the Danish-Swedish Milk Genomics Initiative, morning milk samples were collected from 456 DH cows (20 dairy herds, October - December 2009) and 436 DJ (22 dairy herds, February – April 2010). The overall experimental strategy underlying this study was to minimize the potential sources of environmental variation and maximize the genetic variation in the sample population. From each herd, between 19 and 24 cows were sampled. All cows were housed indoors in loose housing systems, fed according to standard practice, and milked twice a day. The cows sampled were all in mid-lactation (d129 to d229 in DH and d130 to d252 in DJ) and within parity 1 to 3. Immediately after milking, milk samples were placed on ice for transport to the laboratory. Once at the laboratory, the milk samples were aliquoted and skimmed as described [21].

### Fatty acids

The analysis of fatty acid from the milk has previously been described [10]. In short, fat percentage (FP) was determined on fresh whole milk samples by infrared spectroscopy using a MilkoScan FT2 (Foss Analytical, Hilleroed, Denmark). Cream was separated from skim milk by centrifugation (2,643 × *g* for 30 min at 4°C). Hereafter, the cream samples were stored at -20°C until analysis of FA composition using GC, essentially as described by Larsen et al. [22]. The traits used for the association mapping were: C6:0, C8:0, C10:0, C12:0, C13:0, C14:0, C14:1, C15:0, C16:0, C16:1, C18:0, C18:1c9, C18:1 t11, C18:2n6, C18:3n3, C18:2c9t11 (CLA), groups of FA (C6-C10, C6-C12, C12-C14; uneven chain FA were not included), C14 index (C14:1/(C14:1 + C14:0) × 100), C16 index (C16:1/(C16:1 + C16:0) × 100), C18 index (C18:1c9/(C18:1c9 + C18:0) × 100) and CLA index (CLA/(CLA + C18:1 t11) × 100). Poulsen et al. [10] gives an overview of the mean and standard deviation of the fatty acid traits for both the DH and the DJ (Additional file 9: Table S9).

### SNP chip and genotyping

The genotyping procedure has been described in Buitenhuis et al. [23]. In short, 371 DH and 321 DJ cows have been genotyped with the bovineHD beadchip [24]. The bovineHD chip contains 777,962 markers with a median interval of 2.68 kb between SNPs (http://www.illumina.com/documents/products/datasheets/datasheet_bovineHD.pdf). Genomic DNA was extracted from ear tissue. The platform used was an Illumina® Infinium II Multisample assay device. SNP chips were scanned using iScan and analyzed using Beadstudio version 3.1 software. The quality parameters used for the selection of SNPs and animals in the GWAS were minimum call rates of 80% for individuals and 95% for loci. Marker loci with minor allele frequencies (**MAFs**) below 1% were excluded. The quality of the markers was assessed using the GenCall data analysis software of Illumina. Individuals with average GenCall scores below 0.65 were excluded following Teo et al*.* [25]. The SNP positions were based on the *Bos taurus* genome assembly (*Btau_4.0*) [26]. In total 494,984 SNP markers were used in both the DH and DJ.

### SNPs assigned to genes and biological pathways

To assign SNPs to KEGG pathways (http://www.genome.jp/kegg/pathway.html), the KEGG pathways were downloaded using R. The KEGG pathways contain the genes assigned to these pathways. Subsequently, the SNPs on the bovineHD chip were mapped to the UMD3.1 Bovine Genome assembly [27]. Gene positions for this assembly were downloaded September 1st 2011 from ftp://ftp.cbcb.umd.edu/pub/data/assembly/Bos_taurus/Bos_taurus_UMD_3.1/annotation/UMD3.1.gff.gz, containing 26,352 genes with an Entrez Gene ID. Connections between KEGG pathways and Entrez gene IDs were obtained from the BioConductor [28] package ‘org.Bt.eg.db’ v. 2.6.4 (http://www.bioconductor.org/packages/release/data/annotation/html/org.Bt.eg.db.html). A marker was associated maximum once per pathway and associated to a pathway if the marker resided in a gene linked to a pathway.

### Statistical analysis

The statistical analysis was based on the 371 DH and 321 DJ animals having both genotypes and phenotypes.

#### Calculation of the G-matrix

The calculation of the genomic relationship matrix has been described in detail by Buitenhuis et al. [23]. In short, the genomic relationship matrix was calculated for each breed separately. For each chromosome, a genomic relationship matrix as described by the first method presented in VanRaden et al. [29] was calculated as follows: Let **M** be a matrix with dimensions of the number of individuals (*n*) by the number of loci (*m*) that specifies which marker alleles each individual inherited. The elements of **M** were set to -1, 0, 1 for the homozygote, heterozygote and the other homozygote, respectively. The diagonals of **M’M** counts the number of homozygous loci for each individual and off diagonals measure the number of alleles shared by relatives. Let the frequency of the second allele at locus *i* be *p*_*i*_, and let **P** contain the allele frequencies, such that column *i* of **P** equals 2(*p*_*i*_-0.5). Subtraction of **P** from **M** gives **Z**, which is needed to set the expected mean value to 0. The genomic relationship matrix **G** was then calculated as **ZZ´**/[2∑p_i_(1-p_i_)] [29].

#### Association mapping

The analysis was done using the following linear model for each breed separately:
1

Where Y_ijklm_ is the phenotype of individual k in herd i and lactation j, μ is the fixed mean effect, herd_i_ is a fixed effect (i = 1, 2, … , 20 DH; i =1, 2, …, 22 DJ), parity_j_ is a fixed effect (j = 1, 2, 3 DH, j = 1, 2, 3 DJ), b_1_ is the regression coefficient for DIM_k_, DIM_k_ is a covariate of days in milk (d129 to d229 in DH, d130 to d252 in DJ), b_2_ is the allele substitution effect, SNP_l_ is a covariate indicating the if a SNP is homozygote (0,2) or heterozygote (1), and animal is the random additive genetic effect based on **G** of animal k [30]. The effect of the SNP was tested by a Wald test with a null hypothesis of H_0_: b = 0. The analyses were performed using REML in the R interface of DMU [31] (available at http://dmu.agrsci.dk). Significance thresholds were determined using a false discovery rate (FDR) correction using the R package “qvalue” version 1.34.0. A FDR of P < 0.10 was considered significant.

#### Biological pathway analysis

In genome-wide association studies, a large number of correlated tests are routinely performed to detect genetic variants (e.g. SNPs) associated to complex trait phenotypes. In order to control the probability of false discoveries, it is necessary to use multiple-comparison methods such as Bonferroni or FDR to adjust for the large number of test. These corrections greatly reduce the power to detect true associations, which means that genetic variants with small or intermediate effects remain undiscovered. To alleviate this major problem, we applied a biological pathway method that reduces the number of tests from more than 400 k (number of SNPs) to about 200 (number of pathways) using a priori biological information. We do this by grouping SNPs according to their physical location within annotated genes and how these genes are connected in biological pathways (KEGG). For each set we construct an appropriate summary statistics that characterizes the set. We used a summary statistic to identify genes that can explain the phenotypic variation within the traits [32]. It is based on the minus of the log-transformed p-values from the association of individual genetic variants to the phenotypes using the Wald’s test described above. By summing these values we imitate a genetic model capturing variants with small to moderate effects [33, 34]. Using a permutation approach the observed summary statistic for a particular SNP set is compared to an empirical distribution for the summary statistics of random samples of SNP sets of same size. Consider a vector of test statistics, one for each SNP tested in the single-variant approach, ordered after their physical position of the SNP on the genome. As a consequence of linkage disequilibrium closely linked SNPs will likely be correlated, which will affect the distribution of the summary statistics, thus to account for this correlation structure we used the following procedure, Figure 1. Let the vector of observed test statistics be ordered accordingly to the physical position on the genome of the corresponding SNPs. SNPs are then mapped to genes using the coordinates for the physical location of the genes on the genome. Let the elements in this vector be numbered *1*, *2*, …, *N*. The permutation consists of two steps. 1) Randomly pick an element (*e*_*j*_) from this vector. Let this *j*^th^ test statistic be the first element in the permuted vector and the remaining elements ordered *e*_*j+1*_, *e*_*j+2*_, …, *e*_*N*_, *e*_*1*_, *e*_*2*_, …, *e*_*j-1*_ accordingly to their original numbering. Thus, all elements from the original vector are now shifted to a new position starting with *e*_*j*_. The mapping of genes is however kept fixed as to the original mapping. 2) A summary statistic is computed for each SNP set based on the original SNP set position in the original vector of test statistics. Hereby the link between SNPs and genes are broken while retaining the correlation structure among test statistics. Step 1 and 2 are repeated 10,000 times and from this empirical distribution of summary test statistics for each SNP set a *P-*value can be obtained. This empirical *P-*value corresponds to a one-side test of the proportion of randomly sampled summary statistics that are larger than the observed summary statistic. The arbitrary significance level was set to 0.01.

#### Enrichment analysis

The overlap of the associated SNPs in the two breeds was compared within each trait. This was done by constructing for each breed an incidence matrix with n = 494,984 rows corresponding to the number of SNPs and m = 25 columns corresponding to the number of traits. For each breed if the test statistic for a SNP was above the significance threshold then the corresponding element in the incidence matrix was set to one and otherwise zero. The observed overlap in the associated SNPs in the two breeds for each trait was computed based on the matrix product between the two incidence matrices. This overlap was compared to an empirical distribution of the overlap as follows. For a total of 1,000 times the element within each column (i.e. trait) was permutated using the same procedure as described above, and the overlap among columns was recorded. The probability of the found overlap was estimated under the null hypothesis of independence of association among traits. We determined the empirical P-value of a one-sided test as the fraction of all random permutations that was larger or equal than the observed overlap among columns (i.e. traits).

### Availability of supporting data

No new SNPs were discovered in this manuscript. The SNPs used in this manuscript are from the Illumina Bovine HD SNP array: http://res.illumina.com/documents/products/datasheets/datasheet_bovinehd.pdf. Names and location of these SNPs can be found at: http://support.illumina.com/downloads.html. Assigning the SNPs to genes was based on the publicly available bovine genome assembly: ftp://ftp.cbcb.umd.edu/pub/data/assembly/Bos_taurus/Bos_taurus_UMD_3.1/annotation/UMD3.1.gff.gz .

## Electronic supplementary material

Additional file 1: Table S1: Significant SNP associated to Fatty Acid traits in the Danish Holstein. A genome wide association scan was performed on the Danish Holstein cattle for detailed fatty acid composition. SNPs were considered significant at FDR < 0.10. (TXT 190 KB)

Additional file 2: Table S2: Significant SNP associated to Fatty Acid traits in the Danish Jersey. A genome wide association scan was performed on the Danish Jersey cattle for detailed fatty acid composition. SNPs were considered significant at FDR < 0.10. (TXT 174 KB)

Additional file 3: Table S3: Results for the biological KEGG pathway analysis for the individual fatty acids and groups of fatty acids for both the Danish Holstein and Danish Jersey breed. In the table the P-value is given of the one-sided test for overlapping SNPs after 1,000 permutations. (TXT 58 KB)

Additional file 4: Table S4: Significant (P < 0.05) results for the biological KEGG pathway analysis for fat percentage for both the Danish Holstein and Danish Jersey breed. (TXT 3 KB)

Additional file 5: Table S5: Significant (P < 0.05) results for the biological KEGG pathway analysis for C14 index for both the Danish Holstein and Danish Jersey breed. (TXT 3 KB)

Additional file 6: Table S6: Significant (P < 0.05) results for the biological KEGG pathway analysis for C14:1 for both the Danish Holstein and Danish Jersey breed. (TXT 3 KB)

Additional file 7: Table S7: Significant (P < 0.05) results for the biological KEGG pathway analysis for C16 index for both the Danish Holstein and Danish Jersey breed. (TXT 4 KB)

Additional file 8: Table S8: Significant (P < 0.05) results for the biological KEGG pathway analysis for C16:1 for both the Danish Holstein and Danish Jersey breed. (TXT 3 KB)

Additional file 9: Table S9: Mean and standard deviation for the fatty acids for both the Danish Holstein and Danish Jersey breed (Table modified from Poulsen et al. [10]). (TXT 759 bytes)
